# Determination, speciation and distribution of mercury in soil in the surroundings of a former chlor-alkali plant: assessment of sequential extraction procedure and analytical technique

**DOI:** 10.1186/1752-153X-7-178

**Published:** 2013-11-19

**Authors:** Tiberiu Frentiu, Bogdan Petru Pintican, Sanziana Butaciu, Alin Ironim Mihaltan, Michaela Ponta, Maria Frentiu

**Affiliations:** 1 Faculty of Chemistry and Chemical Engineering, Babes-Bolyai University, 11 Arany Janos, 400028, Cluj-Napoca, Romania; 2 National Institute for Research and Development of Optoelectronics Bucharest, Research Institute for Analytical Instrumentation, Donath 67, 400293, Cluj-Napoca, Romania

**Keywords:** Mercury determination, Mercury speciation, Principal component analysis, Custer analysis, Capacitively coupled plasma microtorch

## Abstract

**Background:**

The paper presents the evaluation of soil contamination with total, water-available, mobile, semi-mobile and non-mobile Hg fractions in the surroundings of a former chlor-alkali plant in connection with several chemical soil characteristics. Principal Component Analysis and Cluster Analysis were used to evaluate the chemical composition variability of soil and factors influencing the fate of Hg in such areas. The sequential extraction EPA 3200-Method and the determination technique based on capacitively coupled microplasma optical emission spectrometry were checked.

**Results:**

A case study was conducted in the Turda town, Romania. The results revealed a high contamination with Hg in the area of the former chlor-alkali plant and waste landfills, where soils were categorized as hazardous waste. The weight of the Hg fractions decreased in the order semi-mobile > non-mobile > mobile > water leachable. Principal Component Analysis revealed 7 factors describing chemical composition variability of soil, of which 3 attributed to Hg species. Total Hg, semi-mobile, non-mobile and mobile fractions were observed to have a strong influence, while the water leachable fraction a weak influence. The two-dimensional plot of PCs highlighted 3 groups of sites according to the Hg contamination factor. The statistical approach has shown that the Hg fate in soil is dependent on pH, content of organic matter, Ca, Fe, Mn, Cu and SO_4_^2-^ rather than natural components, such as aluminosilicates. Cluster analysis of soil characteristics revealed 3 clusters, one of which including Hg species. Soil contamination with Cu as sulfate and Zn as nitrate was also observed.

**Conclusions:**

The approach based on speciation and statistical interpretation of data developed in this study could be useful in the investigation of other chlor-alkali contaminated areas. According to the Bland and Altman test the 3-step sequential extraction scheme is suitable for Hg speciation in soil, while the used determination method of Hg is appropriate.

## Background

Mercury is considered one of the most toxic elements in almost all forms even in low concentrations as a result of bioavailability, mobility and high bioaccumulation factor (biomagnification factor up to 10^6^ in the food chain) [1-3]. For this reason its determination and speciation is of great interest in all environmental compartments, such as soil, airborne particulate matter and dust, sediment, water, waste, air and biological samples [4-10]. Natural sources of Hg emission account for 5207 Mg yr^-1^, while the anthropogenic contribution is estimated to account for 2320 Mg yr^-1^, of which more than 95% has been released during the last century [11,12]. The main natural sources of Hg relate to evasion from marine surface waters, biomass burning and volcanoes emission making Hg contamination a global concern. The anthropogenic occurrence of Hg from local sources can create hotspots as is the case of fossil-fuel fired power plants and recently biocombustible, mining (cinnabar, gold, polymetallic ores), processing of non-ferrous metals, cement plants, municipal and medical waste incinerators, chlor-alkali facilities. The diffuse anthropogenic sources of Hg include traffic, human crematories, biomass and coal burning for domestic heating and uncollected waste products (fluorescent lamps, batteries, thermometers, non- and biodegradable packaging materials, etc.). As a result, the studies related to total Hg concentration in soil and plants, as well as the distribution of its species in soil nearby chlor-alkali facilities [13], cinnabar mine [14,15], urbane areas [16], agricultural and forest zones [17] are of great interest. Only monitoring total Hg in environment gives limited data and speciation analysis is mandatory as it provides more useful information related to anthropogenic sources, distribution of Hg forms, potential toxicity and health risk. The non-chromatographic methods are useful tools in environmental studies for providing operationally-defined fractionation of Hg species following single or sequential extraction in specific reagents [13-15,18,19].

Determination of Hg in environmental solid samples involves cold vapor (CV) generation from digested samples in acidic media and detection by atomic fluorescence spectrometry (CV-AFS) and optical emission spectrometry or mass spectrometry in inductively coupled plasma (CV-ICP-OES, CV-ICP-MS) [13,20-22]. Alternatives to these conventional methods are direct release of Hg vapor by thermal desorption from solid sample and detection by atomic absorption spectrometry [4,14,16]. The development of methods for Hg determination meeting green analytical chemistry demands such as microplasma sources/microtorches of low power and low Ar consumption equipped with microspectrometers has became in recent years an innovative field [23-25]. In line with this trend a miniature equipment with a capacitively coupled plasma microtorch and detection by optical emission spectrometry was developed in our laboratory and successfully applied for Hg determination in different materials after digestion and CV generation (CV-μCCP-OES) [26,27]. Recently it has been demonstrated that the analytical technique based on CV-μCCP-OES provides figures of merit for Hg determination in soil similar to the standardized CV-AFS [28].

The aim of this study was the assessment of soil contamination with Hg by determining total content as well as water-available, mobile, semi-mobile and non-mobile fractions of Hg in samples collected from an area under the influence of a former chlor-alkali plant in Romania.

### Experimental

### Site description and sample collection

The case study refers to the Turda town, a former industrial center in north-western Romania. The local economy was based mainly on chemical industry, building materials (cement), glass, porcelain and metallurgy. The Turda Chemical Plant founded in 1911 and closed more that 15 years ago generated an important contamination of soil with Hg from chlor-alkali electrolysis, but also with other metals such as Cu and Zn. The manufactured compounds were sodium hydroxide, chlorine, hydrochloric acid, copper pesticides, Fe, Zn, Na and K salts, and Ca hypochlorite. After 1998 the industrial facilities were closed and partially demolished. No measures were undertaken for soil remediation so that Hg has remained a pollutant of concern in the area of the former chemical plant and perhaps also in the residential zone. Currently, the cement factory is also closed and only a distribution unit has remained in the zone. Several existing manufacturing units related to gypsum cardboard and adhesives do not represent pollution sources.

A number of 38 soil samples were collected from a depth between 20 and 30 cm during May 2013 from the perimeter of the former chlor-alkali plant (7) and waste landfills (5), and residential area (26). Samples were transported in polyethylene bags to laboratory.

### Reagents, standard solutions and CRMs

Nitric acid, 65% ultrapure, hydrochloric acid, 37% ultrapure, sulfuric acid 98%, ultrapure and ethanol for chromatography (Merck, Darmstadt, Germany) were used for soil sample preparation. Standard solution of 1000 mg/l Hg (Merck, Darmstadt, Germany) was used to prepare working standards in the range 0.1 – 10 ng/ml stabilized in 5% (v/v) HCl. Stannous chloride dihydrate for mercury determination (Merck, Darmstadt, Germany) served to prepare 20% (w/v) SnCl_2_ in 15% (v/v) HCl as derivatization reagent. The BrCl solution prepared by dissolution of 1.50 g KBr (99.9 +% p.a.) and 1.08 g KBrO_3_ (99.9 +% p.a.) in 100 ml concentrated HCl was used for oxidation of the organic matter in aqueous extracts, while the 12% (w/v) hydroxylamine in water for reducing the excess of BrCl. The ICP multielement standard solution IV 1000 mg/l (Merck, Darmstadt, Germany) was used to prepare multielement working standards in the range 0 – 25 μg/ml by dilution with 2% HNO_3_ necessary in the determination of Al, Ba, Ca, Cr, Cu, Fe, K, Li, Mg, Mn, Na, Sr and Zn by ICP-OES. Solutions of 0.1 N K_2_Cr_2_O_7_ and 0.2 N Mohr΄s salt were prepared for the determination of organic matter. Solutions necessary for the determination of leachable content of Cl^-^, NO_3_^-^ and SO_4_^2-^ by high performance liquid chromatography were prepared according to SR ISO 10304-1:2007. All solutions were prepared with Milli-Q (18 MΩ/cm) water obtained in laboratory (Millipore Corp., Bedford, USA).

Four certified reference materials, RTC-CRM048-50G Trace Metals Sand 1, RTC-CRM025-050 Soil (Sandy loam-Metals), LGC6141 Soil contaminated with clinker ash and LGC6135 Soil-Hackney Brick Works (LGC Promochem, Wesel, Germany), were analyzed to check the accuracy of the Hg measurements by CV-μCCP-OES.

### Soil sample preparation and characterization

Besides Hg, other soil chemical characteristics (pH, organic matter, total content of 13 metals and water leachable content of Cl^-^, NO_3_^-^ and SO_4_^2-^) were considered. The pollution with Hg was assessed by soil ranking on different categories related to the alert, intervention levels and contamination factor. Considering the contaminated soil as waste a classification on three categories was also made based on the leachability assay.

Samples were dried at room temperature to avoid Hg loss and the soil moisture was determined on a parallel sample at 105 ± 5°C. For the determination of total Hg and the other metals the soil samples were mineralized with aqua regia as specified in SR ISO 11466:1995. An amount of 250 mg test soil sample ground and sieved to < 250 μm was subjected to microwave-assisted digestion with 12 ml aqua regia using the program given in ref. [28]. The digest was filtered and diluted to 100 ml ultrapure water. Soil samples were subjected to water leaching following the procedure SR ISO 12457-1:2003 at a liquid-to-solid ratio of 2:1 to determine the Hg available fraction and concentration of anions (Cl^-^, NO_3_^-^, SO_4_^2-^). An amount of wet sample sieved through the 4 mm sieve corresponding to 175 g dry sample was leached in the REAX 20 shaker (Heidolph, Schwabach, Germany) for 24 ± 0.5 h at room temperature (20 ± 5ºC) with a volume of water corresponding to a liquid-to-solid ratio of 2:1. Mercury species fractionation as: (i) mobile; (ii) semi-mobile and (iii) non-mobile involved a 3-step sequential extraction according to EPA 3200 scheme [19]. The mobile fraction contains organic (CH_3_HgCl) and inorganic Hg^2+^ species (chloride, nitrate, sulfate, oxide and hydroxide). The semi-mobile Hg species in soil relates mainly to elemental Hg and possibly amalgams, while the non-mobile fraction to sulfide (HgS) and calomel (Hg_2_Cl_2_).

*Step 1. Mobile Hg species fraction*: 1.5 g test soil sample was subjected to 3×2.5 ml 2% (v/v) HCl and 10% (v/v) ethanol ultrasound assisted extraction at 60 ± 2°C for 7 min each time. After each extraction the supernatant was separated by centrifugation at 3100 rpm for 5 minutes. After the last extraction the residue was washed with 2.5 ml ultrapure water by manual shaking for 1 min, then the supernatant was separated by centrifugation. The extracts and rinse were combined.

*Step 2. Semi-mobile Hg species fraction*: the residue from the first step was washed with portions of 5 ml warm water of 60 ± 2°C in the ultrasound bath for 5 min each time until the supernatant was free of Cl^-^. Rinses were discarded. Extraction was performed twice with 5 ml 1:2 HNO_3_ on water bath at 95 ± 2°C for 20 min. The residue was rinsed with 5 ml ultrapure water in ultrasound bath for 1 min. Extracts and rinse were combined.

*Step 3. Non-mobile Hg species fraction*: The residue from the previous step was extracted twice with 5 ml 1:6:7 (v/v/v) HCl:HNO_3_:H_2_O for 20 min at 95 ± 2°C on water bath. Supernatants were separated by centrifugation, while the residue was washed by manual shaking with 5 ml ultrapure water for 1 min. Extracts and rinse were combined.

Appropriate aliquot volumes from each extract were subjected to oxidation with 500 μl BrCl solution and the excess was reduced with 500 μl 12% hydroxylamine. Then 2.5 ml concentrated HCl were added and the sample was diluted to 50 ml with water.

For the determination of organic matter an amount of 0.5 g soil was oxidized at 100°C with 10 ml 0.1 N K_2_Cr_2_O_7_ solution in the presence of 20 ml 98% H_2_SO_4_. After cooling, the excess of K_2_Cr_2_O_7_ was back titrated with a solution of 0.2 N Mohr΄s salt in the presence of o-phenanthroline as indicator [29].

For the determination of soil pH a 1:5 (volume fraction) suspension of soil in water was prepared according to ISO 10390:2005.

The concentrations of anions in the water leachate were determined following the procedure given in ISO 10304-1:2007 for water quality control.

## Methods

### Soil analysis

**Figure 1:**
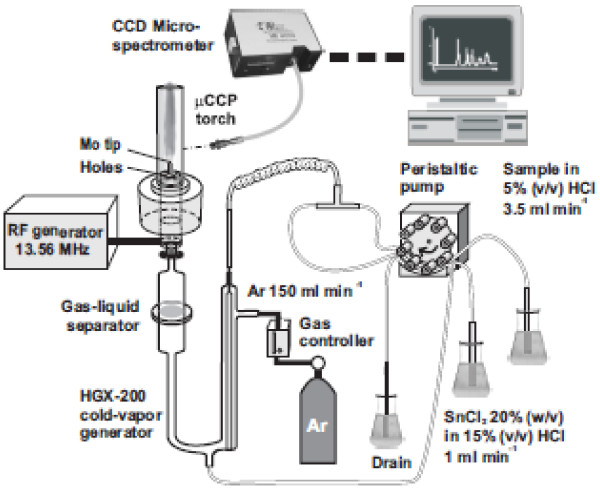
Schematic of the CV-μCCP-OES experimental set-up for Hg determination in soil.

The CV-μCCP-OES analytical system used for Hg determination (Figure 1) consists of a capacitively coupled plasma microtorch, a radiofrequency generator as plasma power source, an Ocean Optics QE65 Pro microspectrometer and an HGX-200 cold vapor generator. The main characteristics and operating conditions of the CV-μCCP-OES system are presented in Additional file 1. The method is based on the derivatization of Hg^2+^ from aqueous solutions to Hg cold vapor following mixing sample solution and SnCl_2_ acidic solution in the cold vapor generator. The Hg vapor are purged from solution *via* an Ar flow (150 ml/min) and introduced into the plasma microtorch. The Hg emission is measured at 253.652 nm. Constructive details related to plasma microtorch and optimal conditions for Hg signal measurement using the CV-μCCP-OES system were previously presented [27,28]. The method detection limit (3σ concept) was 4.8 μg/kg Hg, while the practical quantification limit (PQL) in soil samples 14.4 μg/kg [28], 7 times lower than 100 μg/kg, considered as the normal level in soil.

Total Al, Ba, Ca, Cr, Cu, Fe, K, Li, Mg, Mn, Na, Sr and Zn concentrations were determined by ICP-OES using a SpectroCiros^CCD^ instrument (Kleve, Germany), while water leachable concentration of Cl^-^, NO_3_^-^ and SO_4_^2-^ by high performance ion chromatography using a 761 Compact IC Metrohm (Herisau, Switzerland). The pH of soil was measured with a 350i Multiparameter (WTW, Wilheim, Germany) in 1:5 suspension soil:water.

The results obtained in the determination and speciation of Hg are presented in Table 1, while the chemical composition of soil in Additional file 2.

**Table 1:** Content (mg/kg) of total mercury, water leachable, mobile, semimobile and non-mobile fractions

**Sample**	**Total mercury in aqua regia**		**Water leachable**		**Mobile fraction**		**Semimobile fraction**		**Non-mobile fraction**		**Fraction sum**	
	**Content**^**a**^	**s**_**r**_^**a,b**^	**Content**^**a**^	**s**_**r**_^**a,b**^	**Content**^**a**^	**s**_**r**_^**a,b**^	**Content**^**a**^	**s**_**r**_^**a,b**^	**Content**^**a**^	**s**_**r**_^**a,b**^	**Content**^**c**^	**s**_**r**_^**d**^
1	0.72	0.01	0.008	0.001	0.034	0.001	0.39	0.01	0.23	0.01	0.65	0.01
2	0.095	0.006	0.005	0.001	0.008	0.001	0.083	0.002	0.003	0.001	0.094	0.002
3	0.46	0.01	0.008	0.001	0.040	0.001	0.26	0.01	0.12	0.01	0.42	0.01
4	0.47	0.01	0.013	0.001	0.053	0.002	0.39	0.01	0.041	0.001	0.48	0.01
5	0.24	0.01	0.007	0.001	0.012	0.001	0.22	0.01	0.022	0.002	0.25	0.01
6	1.99	0.02	0.0009	0.0001	0.070	0.002	1.42	0.05	0.57	0.01	2.06	0.05
7	0.13	0.01	0.0090	0.0003	0.022	0.002	0.061	0.001	0.043	0.001	0.13	0.01
8	0.67	0.01	0.0030	0.0001	0.0090	0.0003	0.57	0.03	0.073	0.002	0.65	0.03
9	1.27	0.02	0.0060	0.0003	0.094	0.006	0.79	0.02	0.37	0.01	1.25	0.02
10	0.45	0.01	0.0070	0.0003	0.0080	0.0001	0.34	0.01	0.041	0.005	0.39	0.01
11	0.23	0.01	0.0003	0.0001	0.028	0.002	0.17	0.01	0.035	0.001	0.23	0.01
12	0.38	0.01	0.0060	0.0002	0.019	0.001	0.33	0.01	0.022	0.001	0.37	0.01
13	0.35	0.01	0.0090	0.0004	0.024	0.001	0.25	0.01	0.12	0.01	0.39	0.01
14	0.075	0.004	0.0040	0.0002	0.018	0.001	0.034	0.001	0.036	0.001	0.088	0.002
15	0.42	0.01	0.0040	0.0002	0.022	0.001	0.25	0.01	0.14	0.01	0.41	0.01
16	85.5	0.6	0.017	0.001	5.60	0.04	65.6	0.5	20.2	0.1	91.4	0.5
17	92.6	1.7	0.025	0.002	31.20	1.00	33.3	0.2	20.0	0.3	84.5	1.1
18	26.3	0.3	0.37	0.02	4.78	0.16	16.7	0.2	5.20	0.02	26.7	0.3
19	19.3	0.2	0.0070	0.0004	0.85	0.02	20.1	0.3	1.79	0.01	22.7	0.3
20	114	1	0.051	0.002	7.80	0.02	104	1	1.76	0.04	114	1
21	35.9	2.2	0.019	0.002	0.22	0.01	42.7	2.0	0.50	0.01	43.4	2.0
22	54.8	0.5	1.20	0.06	9.31	0.29	31.3	0.6	6.18	0.32	46.8	0.7
23	0.57	0.02	0.011	0.001	0.017	0.001	0.37	0.037	0.067	0.001	0.45	0.04
24	0.079	0.011	0.007	0.001	0.015	0.001	0.055	0.001	0.015	0.001	0.085	0.002
25	16.8	0.1	0.089	0.009	0.54	0.03	15.5	0.2	2.92	0.01	19.0	0.2
26	0.70	0.01	0.0040	0.0003	0.020	0.001	0.57	0.01	0.15	0.01	0.74	0.01
27	0.28	0.01	0.022	0.003	0.036	0.001	0.23	0.01	0.032	0.001	0.30	0.01
28	0.20	0.01	0.0060	0.0003	0.017	0.001	0.12	0.01	0.090	0.001	0.23	0.01
29	4.44	0.01	0.097	0.004	0.21	0.01	3.27	0.01	0.24	0.01	3.72	0.02
30	0.41	0.01	0.011	0.001	0.012	0.001	0.36	0.01	0.046	0.003	0.42	0.01
31	0.12	0.01	0.0030	0.0002	0.021	0.001	0.069	0.001	0.035	0.001	0.13	0.01
32	0.42	0.01	0.0070	0.0002	0.021	0.001	0.15	0.01	0.16	0.01	0.33	0.01
33	0.11	0.01	0.0040	0.0001	0.014	0.001	0.072	0.001	0.021	0.001	0.11	0.01
34	0.84	0.01	0.0050	0.0005	0.098	0.002	0.48	0.01	0.24	0.01	0.82	0.01
35	20.0	0.31	0.013	0.002	0.81	0.01	18.1	0.3	0.64	0.01	19.6	0.30
36	6.46	0.10	0.013	0.001	0.53	0.02	4.87	0.39	0.45	0.01	5.85	0.30
37	8.88	0.05	0.0010	0.0001	0.39	0.02	7.80	0.06	1.00	0.01	9.19	0.06
38	0.62	0.03	0.0070	0.0003	0.042	0.002	0.51	0.01	0.12	0.01	0.67	0.01
**Mean**	**13.1**		**0.055**		**1.66**		**9.78**		**1.68**		**13.1**	
**Variance**	**27.8**		**0.20**		**5.38**		**21.3**		**4.60**		**27.6**	
**Median**	**0.60**		**0.007**		**0.035**		**0.39**		**0.13**		**0.57**	
**Min**	**0.08**		**0.0003**		**0.008**		**0.034**		**0.003**		**0.085**	
**Max**	**114**		**1.20**		**31.2**		**104**		**20.2**		**114**	

^a^–n=3 complete dissolution/analysis sequences for each sample.

^b^–standard deviation of repeatability.

^c^–sum of mobile, semi-mobile and non-mobile fractions.

^d^–pooled standard deviation of sum s_pooled_ = (s_1_^2^ + s_2_^2^ + s_3_^2^)^1/2^.

### Statistical analysis

Statistical data processing was carried out with XLStat Microsoft Excel plug-in (Addinsoft). The distribution maps of the total Hg and its species in soil were plotted to identify differences between contamination in the impact zone and surroundings. Principal Component Analysis (PCA) and Cluster Analysis (CA) were used to describe the variability in the chemical composition in the soil samples and identify Hg species of main contribution to the total variability. Principal Component Analysis (PCA) and Cluster Analysis (CA) have been widely used in environmental studies to differentiate between natural and anthropogenic origin of contaminants [30,31]. R-mode PCA was applied to assess the weight of the different fractions of Hg, total concentrations of Hg, Al, Ba, Ca, Cr, Cu, Fe, K, Li, Mg, Mn, Na, Sr, Zn, water leachable concentration of anions (Cl^-^, SO_4_^2-^ and NO_3_^-^), organic matter and pH on the variability of chemical composition of soil and fate of Hg. According to the Kaiser criterion only the PC’s with eigenvalue >1.0 was retained and subjected to varimax rotation. Factor loadings were classified as ‘strong’, ‘moderate’ and ‘weak’ corresponding to absolute loading values of >0.75, 0.50-0.75 and in the range 0.30-0.50, respectively [32]. Cluster Analysis using the Ward’s linkage method and Euclidian distances as a measure of similarity was used to group chemical parameters into classes. The Shapiro-Wilk test was used to verify the null hypothesis in relation with the normal distribution of the parameters under study. The Bland and Altman test [28,33] was applied to assess the 3-step sequential extraction of Hg from soil. Thus the differences (bias) between the sum of the three fractions of Hg species and Hg concentration extracted in aqua regia were plotted versus the mean of results. According to the Bland and Altman test there is no significant bias between two sets of data if the confidence interval of the mean difference contains the zero value and the difference between measurements for each sample lies between the limits of agreement of the results.

### Assessment of the fractionation scheme of Hg species in soil and CV-μCCP-OES analytical technique

**Figure 2:**
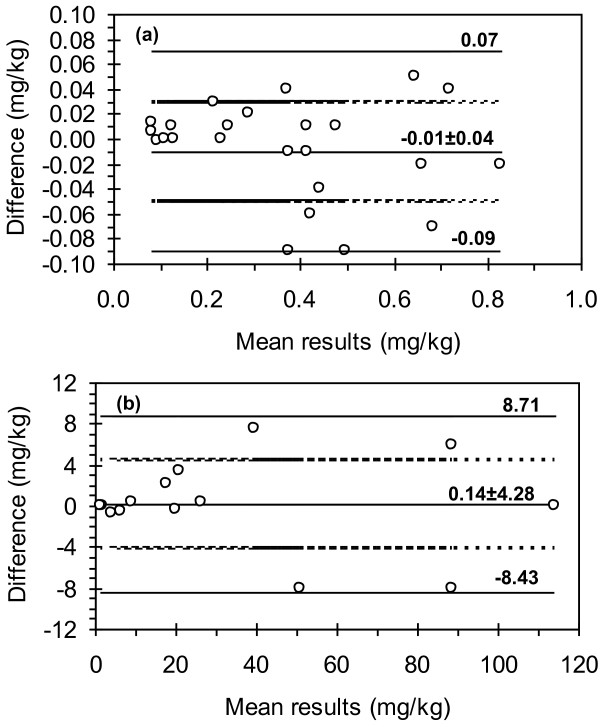
Bland and Altman plots: (a) <1 mg/kg Hg (n=24 samples); (b) > 1 mg/kg Hg (n=14 samples).

The Bland and Altman plots for Hg content < 1 mg/kg (24 samples) and > 1 mg/kg (14 samples) are given in Figure 2. For both concentration ranges the confidence interval of the negative/positive bias towards the sequential extraction included the zero value and the differences between all pairs of data were within the lower and upper limit of agreement. This has demonstrated that the differences between results are random and the 3-step sequential extraction performed in this study is suitable for Hg speciation in soil. The average recovery of Hg using the sequential extraction was 100.5 ± 3.3% for 95% confidence interval related to Hg determined in aqua regia.

Accuracy of Hg determination by the CV-μCCP-OES method was checked by analyzing certified reference materials (CRMs). The results obtained for the analysis of four CRMs of soil (Table 2) show a recovery of 98.3 ± 3.5% for Hg determined in soil by the CV-μCCP-OES technique.

**Table 2:** Results obtained for Hg determination (mg/kg) in certified reference materials by CV-μCCP-OES

**Reference material**	**Certified value ± U**^**a**^	**Found value ± U**^**a **^**(n = 5)**
RTC-CRM048-50G	28.00 ± 1.13	27.50 ± 1.05
LGC 6135	3.2 ± 0.4	3.0 ± 0.1
RTC-CRM 025-050	99.8 ± 31.7	96.7 ± 0.8
LGC 6141	1.20^b^	1.25 ± 0.06

^a^ Expanded uncertainty for 95% confidence interval.

^b^ Indicative value.

## Results and discussion

### Total Hg concentration and distribution of fractions

The content of Hg extracted in aqua regia considered as total, that transferred to water as leachable portion as well as that distributed among the three fractions (mobile, semi-mobile and non-mobile) according to the 3-step extraction scheme are presented in Table 1. As shown in Table 1, the total Hg was in the range 0.074 – 114 mg/kg, while the water leached fraction was much lower, of 0.0003 – 1.20 mg/kg corresponding to 0.01 – 8.8% of the total. The amount of the mobile fraction was between 0.008 – 31.2 mg/kg, accounting for 0.5 – 36.9% of the total three fractions. The semi-mobile fraction (0.034 – 104 mg/kg Hg) attributed mainly to Hg^0^ was the major one accounting for 38.6 – 98.4%. The non-mobile fraction was in the range 0.003 – 20.2 mg/kg corresponding to 1.2 – 48.5% of the sum of fractions. Shortly, the weight of the three fractions increased in the order mobile < non-mobile < semi-mobile. The pattern is consistent with the low solubility of non-mobile species and low reactivity of Hg^0^[34].

The low water-soluble fraction, comparable to that of 0 – 2.7 mg/kg (up to 0.8% of the total Hg) reported in the surroundings of a chlor-alkali plant in Netherlands by Bernaus et al. [13] indicated a poor availability of Hg under weathering processes. The proportion of mobile Hg species according to EPA-3200 method was also found to be close to values reported by Bernaus et al. (5.8 – 18.8% of the total) under alkaline conditions (pH = 8.4) [13], but much out the range of 0.008 – 0.038 mg/kg (0.002 – 0.2% of the total) extracted in 1 M NH_4_NO_3_ by Garcia-Sanchez et al. [14]. On the other side the semi-mobile Hg fraction in soil found by us was much higher compared to the concentrations and weight reported by Bernaus et al. [13] around a chlor-alkali plant, Garcia-Sanchez et al.[14] and Malferrari et al. [15] in soil around an abandoned cinnabar mining area. Our results are rather close to those of Kocman et al. [35], who reported a major fraction (30 – 60%) of Hg^0^ around a cinnabar mine and ore smelting plants. The high semi-mobile Hg fraction in soil in the zone under our study was attributed to anthropogenic input as Hg^0^ of low reactivity and difficult to be converted into water available, mobile or non-mobile species. In fact, in some soil samples collected from the former industrial zones the presence of metallic Hg was visually identified. On the other hand Hg^0^ exhibits high volatility at ambient temperature and can be taken up *via* leaves thus increasing the Hg content in biomass. Several studies emphasized this contamination route in plant specimens growing in soil with high Hg^0^ concentration [14,36]. The non-mobile fraction of Hg found by us was lower than that reported by Bernaus et al. [13] who found this fraction as major (36.0-90.7% of the total). Corroboration of our results with literature data suggests that the fraction distribution, fate of anthropogenic Hg and its persistence in soil are governed by industry type and associated species released in the environment.

### Spatial variability of total mercury distribution in soil

**Figure 3:**
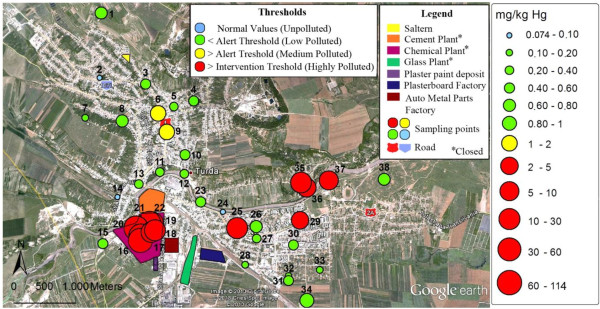
Spatial variability of total Hg in soil and comparison with soil quality guideline (normal content: 0.1 mg/kg; alert threshold: 1 mg/kg and intervention threshold: 2 mg/kg).

In order to highlight the spatial variability of soil contamination, the punctual Hg concentrations were compared to normal content, alert and intervention thresholds for soil in sensitive areas given in the quality guideline [37]. The alert threshold corresponds to the concentration which, if exceeded, imposes an investigation with respect to the individual case in question and is designed to alert authorities about a potential environmental impact. The intervention threshold is related to the concentration of a pollutant which shall enforce risk assessment studies to reduce concentrations by decontamination. The concentrations of total Hg in soil over the entire investigated area were related to reference values for sensitive areas, as all industrial facilities have been closed and soil decontamination is in question in order to return into urban circuit. The analyzed soil samples were classified in four groups based on the Hg contamination factor (C_f_) computed by dividing the Hg concentration by its normal value in soil [30,38]. The spatial variability of total Hg in soil using punctual concentrations and grouping of sites according to C_f_ values are given in Figure 3. Only three sites (2,14 and 24) are classified as non-polluted, while the most sites exhibit a low contamination with C_f_ between 1.1-7.2. The spatial distribution shows that in the area of the former chlor-alkali plant (sites 16-22) the soil is highly contaminated with Hg (C_f_ 193-1136). In the same time, the distribution map reveals an extension of Hg contamination well beyond the area of the former chlor-alkali plant. Thus, elevated Hg contents were found in soil collected from sites 25 (C_f_ 169), 29 (C_f_ 44), 35 – 37 (C_f_ 65 – 200), relatively far from the former pollution source. The contamination of these sites is rather the result of their use as landfills 40-50 years ago than of atmospheric pollution via Hg vapor diffuse transportation by the air currents. Sites 6 and 9 located in the center of the town fall in the category of those medium polluted (C_f_ 13-20) leading to the idea that the influence of urban traffic on soil contamination should also be considered. Anyway, variability of total mercury distribution and high contamination of areas outside the pollution core are consistent with other works [13,14,16] which report large differences among collection points separated by only a few hundred meters.

### Spatial variability of water leachable mercury distribution in soil

**Figure 4:**
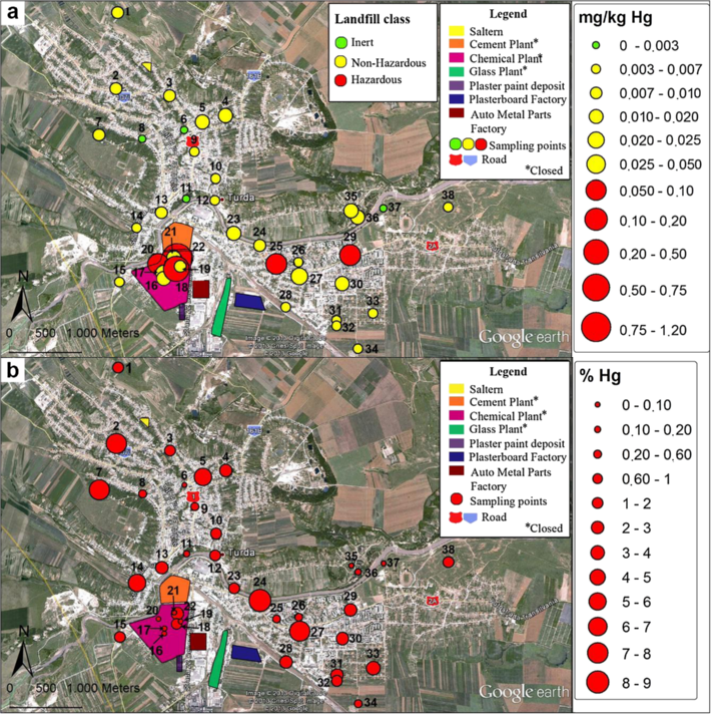
**Spatial distribution of the water leachable Hg. a**. Leached content (mg/kg); **b**. Weight (%) related to total Hg extracted in aqua regia. Comparison with leaching limit values for granular waste (mg/kg): inert <0.003; non-hazardous <0.05 and hazardous > 0. 05.

Besides total mercury, another useful criterion in this study for assessing the potential risk of contamination of the environment was the water available mercury fraction. The reason is that even small concentrations of total mercury in soil may result in a great water mobility posing a risk to the environment. A certain content of water available Hg in soil may qualify it as hazardous waste according [39]. Waste is classified as inert, non-hazardous or hazardous if the Hg water leachable fraction is <0.003 mg/kg, <0.05 mg/kg and > 0.05 mg/kg, respectively. The spatial variability of the water leachable mercury content at a liquid-to-solid ratio of 2 l/kg and grouping of sites according to waste classification criteria are presented in Figure 4a. The weight of this fraction related to that extracted in aqua regia is shown in Figure 4b. The water available fraction increased but generally its weight decreased with total Hg increased. According to Figure 4a, soil in the residential area was found to be non/low polluted in terms of total Hg content and in the same time complied with the leachability limit of non-hazardous waste. Only in 4 collection sites, of which 3 situated in the residential area, soil matched criteria for inert waste with no contamination risk. In samples collected from the industrial area (sites 16-22) and outside the perimeter (sites 25,29) soil was found to be highly polluted in terms of total Hg content and the leachability limit of Hg in hazardous waste was also surpassed. As a consequence the soil in these sites exhibits significant risk of water contamination under certain climatic conditions. On the other hand, the soils from sites 35,36 and 37, found also as strongly contaminated from the point of view of total Hg, exhibited low water leachability of Hg species and were categorized as non-hazardous or even inert waste.

### Spatial variability of mobile, semi-mobile and non-mobile Hg species

**Figure 5:**
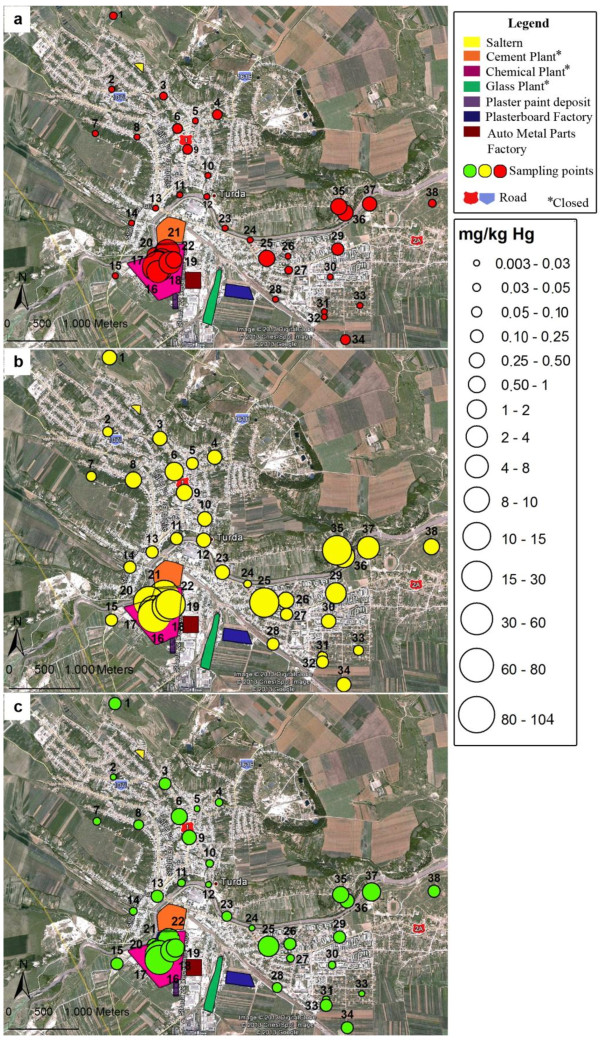
Spatial distribution (mg/kg) of mobile (a), semi-mobile (b) and non-mobile Hg species (c).

**Figure 6:**
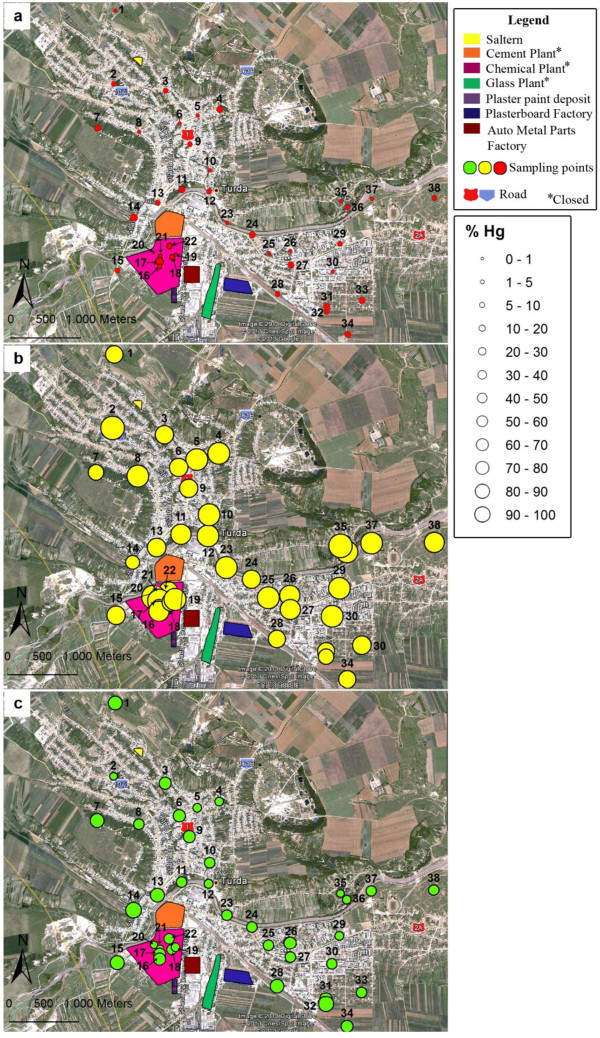
Spatial distribution in weight (%) of mobile (a), semi-mobile (b) and non-mobile Hg species (c) related to the sum of fractions.

The distribution maps of spatial variability of mobile, semi-mobile and non-mobile Hg species concentrations in soil together with the corresponding proportion related to their sum are presented in Figure 5 and Figure 6. Similar patterns with high spatial variability were observed for all three fractions. The measured fractions were the highest in soil with high total Hg content in the former Chemical Plant area as well as in samples collected from sites 25, 29 and 35 – 37. The fraction of semi-mobile Hg species in soil associated mainly to Hg^0^ was dominant throughout the collection area (Figure 5b) with quite uniform distribution in terms of weight of the fractions sum (Figure 6b). This behavior was consistent with the important input of anthropogenic Hg^0^ originating from the industrial zone. The proportion of the semi-mobile fraction decreased on the account of the non-mobile species (Figure 6c) retained by the soil components. Also, the weights of the mobile (Figure 6a) and non-mobile (Figure 6c) Hg fractions were higher in soils from the residential area with low content of total Hg.

### Chemical characterization of soil

Several characteristics of soil besides Hg related to possible contamination in this area are presented in Additional file 2. Some of these parameters could influence the fate of Hg in soil. The pH of soil exhibited a low variability in the slightly basic range (7.7 – 9.3). The content of organic carbon varied from 0.13% (site 20 in the industrial zone) to 2.78% (site 1 outside the town). The concentration of Cr was at the level of the detection limit in ICP-OES (2 mg/kg) in 4 soil samples. The priority hazardous metals (Cd, Pb and Ni) and Co were not taken under consideration for soil characterization as their concentrations were below the detection limits (mg/kg): 1.70 Cd, 50 Pb, 12 Ni and 1.70 Co in any samples. At these concentration levels ICP-OES does not provide the precision necessary for quantification (at least 10%). It has been found a considerable soil contamination in the industrial area (sites 16-22) with Cu and Zn, which exceed occasionally the alert threshold (200 mg/kg Cu and 600 mg/kg Zn) according to [37]. Copper and Zn in these site are of anthropogenic origin associated to production of Cu pesticides and Zn salts. In specific locations, outside the industrial perimeter, the alert threshold for Zn (300 mg/kg) and the intervention threshold for Cu (sites 34-38) were exceeded as the result of the high input of waste deposition in the past. On the other hand, elevated concentrations of K, Ca, Na and Cl^-^ originating from K salt production and calcium hypochlorite produced in the past and NaCl as raw material were found.

### Application of PCA and CA to evaluate the variability of soil chemical composition

Among the parameters used to characterize the chemical composition of soil only pH, total organic carbon (TOC), Ba and Sr exhibited normal distribution according to the Shapiro-Wilk test for 95% confidence level. However the PCA approach is not significantly affected by the lack of normal distribution. The varimax rotated loadings of 7 PC’s with eigenvalue >1 explaining 79.2% of soil chemical variability are presented in Table 3. Mercury or its species as soil characteristics belongs to 3 of the 7 PCs, which totals 41%. The first factor (PC1) accounting for 22.0% of the total variance of soil chemical composition was attributed mainly to total Hg, mobile, semi-mobile and non-mobile Hg fractions with strong influence. Other parameters, like Cu, Fe and SO_4_^2-^ had weak influence. The Hg water leacheable fraction exhibited no influence of PC1 consistent with a different origin from the other Hg species. We may assume that the leachable Hg species exists as CH_3_Hg^+^ produced from inorganic Hg^2+^ species by sulfate reducing bacteria under anoxic condition. The process is facilitated by the increase of the concentration of Hg^2+^ available species as well as of SO_4_^2-^ concentration up to 0.11 g/kg [40]. In the case under study, in sites with high contamination this process is inhibited. Bernaus et al. [13] have found a positive correlation of Hg with Cu and Ni attributed to formation of solid solutions within the same crystalline structure. The correlation of Hg with Cu was also found by us but it was not verified by the X ray diffraction. Similar to Cu, a correlation of Fe with Hg species was identified so that the retention of mobile Hg species by adsorption on Fe oxy-hydroxide could not be neglected as remarked by Kinniburg and Jackson [41]. The presence of SO_4_^2-^ in this PC is consistent with its association with Hg mobile fraction in agreement with the results of Bernaus et al. [13], who reported that in the surroundings of a chlor-alkali plant HgSO_4_ was the main mobile species. Chloride and NO_3_^-^ seem to play no role in the generation of Hg mobile species. The lake of correlation between Hg species on the one hand and some natural components of soil having normal distribution (TOC, pH, Ba and Sr) on the other hand has indicated that the origin of Hg is mainly anthropogenic (Hg^0^). Generally under natural conditions more that 97% of the total Hg in soil is bound to organic matter or precipitated as sulfide [12] but this pattern did not fit to our study. PC2 (15.1%) explains the influence of aluminosilicates on the variability of the chemical composition of soil. The absence of the Hg species in this PC is in agreement with the higher affinity of Hg for acidic minerals compared to basic minerals as demonstrated by Hg determinations in such samples [42].

**Table 3:** Factor loadings after Varimax rotation describing variability of soil chemical composition

	**PC1**	**PC2**	**PC3**	**PC4**	**PC5**	**PC6**	**PC7**
T Hg	0,945	-0,058	0,097	0,082	0,068	0,029	-0,044
L Hg	0,257	-0,096	0,585	-0,026	0,242	-0,244	0,407
M Hg	0,794	-0,028	0,041	0,105	0,041	-0,213	0,096
Sm Hg	0,831	-0,054	0,105	0,076	0,056	0,116	-0,149
N-M Hg	0,839	-0,053	-0,063	-0,023	0,162	0,195	0,173
pH	-0,137	-0,042	0,254	-0,158	-0,155	-0,144	-0,659
TOC	-0,118	0,223	0,129	-0,305	-0,243	-0,112	0,612
Al	-0,216	0,693	-0,323	0,083	-0,093	0,315	0,174
Ba	0,079	0,187	-0,131	-0,088	-0,123	0,846	-0,041
Ca	0,227	0,130	0,860	0,019	0,146	0,045	-0,006
Cr	-0,206	0,030	-0,100	-0,856	-0,196	0,047	-0,043
Cu	0,460	-0,117	-0,009	0,329	-0,009	0,625	0,163
Fe	0,403	0,157	-0,699	-0,195	-0,150	0,288	0,089
K	-0,184	0,805	0,185	-0,227	-0,061	0,158	0,061
Li	0,066	0,731	0,206	0,144	-0,100	0,000	0,114
Mg	-0,015	0,798	-0,237	0,018	-0,047	-0,178	-0,086
Mn	-0,049	-0,057	-0,654	-0,121	-0,151	-0,047	0,137
Na	0,070	-0,020	0,181	0,074	0,946	-0,107	-0,005
Sr	0,075	0,649	0,498	0,345	0,107	0,135	-0,073
Zn	-0,064	0,057	0,102	0,646	-0,151	0,616	-0,065
Cl^-^	0,009	-0,057	0,092	0,053	0,959	-0,132	-0,043
NO_3_^-^	0,023	0,088	0,046	0,860	0,063	0,036	-0,044
SO_4_^2-^	0,458	-0,071	0,017	0,063	0,690	0,326	0,170
Variance (%)	22.0	15.1	14.3	9.7	7.8	5.6	4.7

>0.75 – strong influence; 0.50–0.75 – moderate influence; 0.30–0.50 – weak influence.

T Hg – total Hg extracted in aqua regia; L Hg – water leachable fraction; M Hg – mobile Hg fraction in 2% HCl and 10% ethanol solution; Sm Hg – semi-mobile fraction in 1:2 HNO_3_ solution; N-M Hg – non-mobile fraction in 1:6:7 HCl:HNO_3_:water solution.

Two factors, PC3 (14.1%) and PC7 (4.7%), were assigned to water leachable fraction of Hg. The high positive loading of Ca in PC3 results from an anthropogenic input via Ca hypochlorite produced in the past, which enhances Hg mobility. It is well known the use of Ca hypochlorite for the remediation of Hg contaminated soil [43] but this strategy was not used in the zone studied by us. Iron and Mn as effective sorbents for Hg^2+^ have a moderate negative influence in PC3 on water mobilization of Hg [41].

PC7 (4.8%) shows the influence of two natural characteristics of soil (organic matter and pH) on the chemical composition and water leachable Hg species, and suggests the presence of Hg as organic water soluble species. Their weight tends to increase with organic matter content and to decrease as pH increases.

The forth principal component (PC4) accounting for 9.7% variability reflects a strong influence of Cr as organic species of natural origin and Zn as nitrate of anthropogenic source on the chemical composition of soil. On the other hand these parameters have no influence on Hg species.

The fifth factor (PC5) explaining 7.8% of the total variance was assigned to the influence of Na salts (chloride and sulfate) of anthropogenic origin on the soil chemical composition. The strong factor loadings of Na and Cl^-^ and the moderate one of sulfate suggest the presence of Na mainly as chloride originating from the raw material used in the past. The highest concentrations of these species were found in water leachate of soil from the industrial area. Chloride has no influence on the mobilization of Hg species in agreement with a study of Bernaus et al. [13] who reported a random relationship between Hg and Cl^-^ in soil in the surroundings of a chlor-alkali plant.

PC6 (5.6%) describes the influence of the heavy metals (Cu and Zn) of anthropogenic origin on the chemical composition of soil. The highest concentrations of these metals were found in soil from the perimeter of the former Chemical Plant and in the zone of the former waste landfills. One can observe the positive correlation between these elements and SO_4_^2-^. The coexistence of these elements together with Ba in the same PC has been previously remarked in the case study on river sediment in an area under the impact of non-ferrous ore mining [30].

Two-dimensional plots of the first three PCs made it possible to identify 3 groups of sites on the basis of all parameters under study (Additional file 3). The first group encompasses sites (16-22) in the area of the former Chemical Plant, where soils have been found highly contaminated with Hg and categorized as hazardous waste. To the second group belong sites (35-37) in the area of the former waste landfills also found as highly contaminated with Hg but here the soil was categorized as non-hazardous waste. The third, largest group contains Hg low-contaminated sites where soil corresponds to non-hazardous waste criteria. The same pattern is true considering only the parameters related to Hg species (Additional file 4).

**Figure 7:**
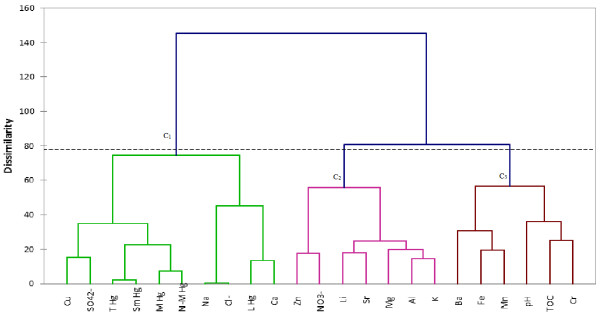
**Dendrogram showing the clustering of soil characteristics.** T Hg – total Hg extracted in aqua regia; L Hg – water leachable fraction; M Hg – mobile Hg fraction in 2% HCl and 10% ethanol solution; Sm Hg – semi-mobile fraction in 1:2 HNO_3_ solution; N-M Hg – non-mobile fraction in 1:6:7 HCl:HNO_3_:water solution.

The dendrogram in Figure 7 presents grouping of soil characteristics in three main clusters. Cluster C1 with 2 sub-clusters contains parameters of dominant influence on soil and confirms the anthropogenic origin of Hg by grouping together total, mobile, semi-mobile and non-mobile Hg species. One can remark the association between Cu and SO_4_^2-^ following the anthropogenic input as CuSO_4_ used as raw material to obtain copper fungicide. The second sub-cluster of C1 indicates the presence of Na as NaCl and the influence of Ca as hypochlorite on water leachability of Hg. The second cluster (C2) groups in two sub-clusters elements usually present in aluminosilicates and Zn as nitrate as previously suggested by PCA. Cluster C3 contains 2 sub-clusters, one of which suggesting Cr binding to organic matter.

## Conclusions

A case study on Hg contamination in the surroundings of a former chlor-alkali plant closed more than 15 years ago was conducted. Determination of total Hg content, water leachable, mobile, semi-mobile and non-mobile Hg species was performed using a novel analytical technique based on CV-μCCP-OES. Results have shown a high spatial variability of contamination with Hg species, of which the Hg^0^ semi-mobile fraction was the main form. The Principal Component Analysis applied on standardized data related to Hg species and chemical composition of soil revealed the main contributors to the variability and the dominant factors governing the fate of Hg species in soil. Total Hg, semi-mobile, non-mobile and mobile species were observed to have the greatest influence on soil chemical composition variability. The influence of the Hg water soluble fraction was weak, although within the perimeter of the former plant and waste landfills soil could be regarded as a hazardous waste according to the test of leachability. PCA also revealed a different origin of the Hg water soluble fraction, mainly bind to organic matter and controlled by soil pH, Ca, Mn and Fe content. Among anions, only sulfate plays a significant role in binding Hg^2+^ species as soluble inorganic compounds. Aluminosilicates, Zn and Cr compounds play no role in the retention of Hg species, while Cu compounds a minor role. Plotting data with respect to the first three PCs is useful to identify the spatial variability of soil contamination with Hg and allows grouping sites with similar contamination factors. Cluster analysis of soil characteristics has confirmed the hypothesis of the anthropogenic origin of Hg in the area contaminated by the chlor-alkali industry by grouping Hg species in the same cluster, distinct from that including natural components of soil such as aluminosilicates. Cluster analysis has also shown soil contamination with Cu as sulfate, Zn as nitrate and the presence of NaCl. The approach based on speciation and statistical interpretation of data such as that used by us in this case study could be of interest to evaluate other areas of similar contamination. The method based on the proposed miniaturized instrumentation has found to be suitable for Hg determination.

## Abbreviations

CV-AFS: Cold vapor atomic fluorescence spectrometry; CV-ICP-OES: Cold vapor inductively coupled plasma optical emission spectrometry; CV-ICP-MS: Cold vapor inductively coupled plasma mass spectrometry; CV-μCCP-OES: Cold vapor capacitively coupled plasma microtorch optical emission spectrometry; CRM: Certified reference material; TOC: Total organic carbon.00000.

## Competing interests

The authors declare that they have no competing interests.

## Authors’ contributions

TF – designed the study and coordinated the preparation of the manuscript, interpreted the results of chemical analysis and multivariate statistical analysis (PCA and CA), and co-worked on the Hg determination by cold vapor capacitively coupled microplasma optical emission spectrometry; MP – co-worked on the soil sample analysis by optical emission spectrometry and performed the data comparison using the Bland and Altman test; AIM – performed soil sample collection, measurements of pH and organic matter and co-worked on sample preparation for Hg determination; BPP – co-worked on sample preparation and analysis by cold vapor capacitively coupled microplasma optical emission spectrometry and prepared the manuscript graphics; SB – co-worked on sample preparation and analysis by cold vapor capacitively coupled microplasma optical emission spectrometry; MF – co-worked on sample preparation and measurements of pH and organic matter in soil samples. All authors read and approved the final manuscript.

## Authors’ information

TF is associate professor of instrumental analysis at the University Babes-Bolyai, Faculty of Chemistry and Chemical Engineering, Cluj-Napoca, Romania. His research field includes the development of analytical methods by optical emission spectrometry in inductively or capacitively coupled plasma sources for the determination of priority hazardous elements in environmental samples and materials. He has also interests in the development of miniaturized analytical instrumentation based on plasma microtorches for on-site analysis.

MP is associate professor of instrumental analysis at the University Babes-Bolyai, Faculty of Chemistry and Chemical Engineering, Cluj-Napoca, Romania. Her area of interests covers the development of analytical methods by atomic spectrometry, toxicological analysis, and quality control and quality assurance in chemical analysis.

AIM is Researcher at Research Institute for Analytical Instrumentation, Cluj-Napoca and PhD student at University Babes-Bolyai, Cluj-Napoca, Romania. His scientific interests relate to monitoring of the priority hazardous metals in environmental samples and different materials, as well as development of analytical methods using instrumentation based on plasma microtorches.

MF is Researcher at Research Institute for Analytical Instrumentation, Cluj-Napoca, Romania. Her scientific interest relates to the development of environmental sample preparation methods.

BPP is engineer and Master student at University Babes-Bolyai, Cluj-Napoca, Romania, with expertise in environmental quality control and depollution techniques.

SB is Master student at the University Babes-Bolyai, Faculty of Chemistry and Chemical Engineering, Cluj-Napoca, Romania.

## Supplementary Material

Additional file 1 Characteristics and working conditions of the CV-μCCP-OES analytical system. Click here for file

Additional file 2 Chemical characterization of soil. Click here for file

Additional file 3 Two dimensional plot of PCs considering all investigated parameters of soil. Click here for file

Additional file 4 Two dimensional plot of PCs considering Hg parameters of soil. Click here for file

## References

[B1] Leopold K Foulkes M Worsfold P Methods for the determination and speciation of mercury in natural waters - a review Anal Chim Acta 2010 663 127 138 10.1016/j.aca.2010.01.048 20206001

[B2] Diez S Bayona JM Determination of Hg and organomercury species following SPME: a review Talanta 2008 77 21 27 10.1016/j.talanta.2008.06.027 18804593

[B3] Nam DH Basu N Rapid methods to detect organic mercury and total selenium in biological samples Chem Cent J 2011 5 3 10.1186/1752-153X-5-3 21232132 PMC3033235

[B4] Kelly JG Han FXX Su Y Xia YJ Philips V Shi ZQ Monts DL Pichardo ST Xia K Rapid determination of mercury in contaminated soil and plant samples using portable mercury direct analyzer without sample preparation, a comparative study Water Air Soil Poll 2012 223 2361 2371 10.1007/s11270-011-1030-3

[B5] Sardans J Montes F Penuelas J Electrothemal atomic absorption spectrometry to determine As, Cd, Cr, Cu, Hg and Pb in soils and sediments: a review and perspectives Soil Sediment Contam 2011 20 447 491 10.1080/15320383.2011.571526

[B6] Guo W Hu SH Wang XJ Zhang JY Jin LL Zhu ZL Zhang HF Application of ion molecule reaction to eliminate WO interference on mercury determination in soil and sediment samples by ICP-MS J Anal At Spectrom 2011 26 1198 1203 10.1039/c1ja00005e

[B7] Araujo RGO Vignola F Castilho INB Borges DLG Welz B Vale MGR Smichowski P Ferreira SLC Becker-Ross H Determination of mercury in airborne particulate matter collected on glass fiber filters using high-resolution continuum source graphite furnace atomic absorption spectrometry and direct solid sampling Spectrochim Acta Part B 2011 66B 378 382

[B8] Leopold K Zierhut A Huber J Ultra-trace determination of mercury in river waters after online UV digestion of humic matter Anal Bioanal Chem 2012 403 2419 2428 10.1007/s00216-012-5851-8 22532060

[B9] Jew AD Kim CS Rytuba JJ Gustin MS Brown GE Jr New technique for quantification of elemental Hg in mine wastes and its implications for mercury evasion into the atmosphere Environ Sci Technol 2011 45 412 417 10.1021/es1023527 21121657 PMC3030447

[B10] Pandey SK Kim KH Brown RJC Measurement techniques for mercury species in ambient air Trends Anal Chem 2011 30 899 917 10.1016/j.trac.2011.01.017

[B11] Pirrone N Cinnirella S Feng X Finkelman RB Friedli HR Leaner J Mason R Mukherjee AB Stracher GB Streets DG Telmer K Global mercury emissions to the atmosphere from anthropogenic and natural sources Atmos Chem Phys 2010 10 5951 5964 10.5194/acp-10-5951-2010

[B12] U.S. EPA Office of Air quality planning & standards and office of research and development, EPA, mercury study: report to congress, Vol III, fate of transport of mercury in the environment, EPA-452/R-97-005 1997 http://nepis.epa.gov/Exe/ZyPURL.cgi?Dockey=2000EICE.txt (accessed 01.05.2013).

[B13] Bernaus A Gaona X van Ree D Valiente M Determination of mercury in polluted soils surrounding a chlor-alkali plant: direct speciation by X-ray absorption spectroscopy techniques and preliminary geochemical characterisation of the area Anal Chim Acta 2006 565 73 80 10.1016/j.aca.2006.02.020

[B14] Garcia-Sanchez A Murciego A Alvarez-Ayuso E Santa Regina I Rodriguez-Gonzalez MA Mercury in soils and plants in an abandoned cinnabar mining area (SW Spain) J Hazard Mater 2009 168 1319 1324 10.1016/j.jhazmat.2009.03.009 19345007

[B15] Malferrari D Brigatti MF Elmi C Laurora A Determination of Hg binding forms in contaminated soils and sediments: state of the art and a case study approaching abandoned mercury mines from Mt. Amiata (Siena, Italy) Neues Jahrbuch Fur Mineralogie-Abhandlungen 2011 188 65 74 10.1127/0077-7757/2011/0194

[B16] Rodrigues S Pereira ME Duarte AC Ajmone-Marsan F Davidson CM Grcman H Hossack I Hursthouse AS Ljung K Martini C Otabbong E Reinoso R Ruiz-Cortes E Urquhart GJ Vrscaj B Mercury in urban soils: a comparison of local spatial variability in six European cities Sci Total Environ 2006 368 926 936 10.1016/j.scitotenv.2006.04.008 16750244

[B17] Tack FMG Vanhaesebroeck T Verloo MG Van Rompaey K Van Ranst E Mercury baseline levels in Flemish soils (Belgium) Environ Pollut 2005 134 173 179 10.1016/j.envpol.2004.05.031 15572235

[B18] Neculita CM Zagury GJ Deschenes L Mercury speciation in highly contaminated soils from chlor-alkali plants using chemical extractions J Environ Qual 2005 34 255 262 15647556

[B19] EPA 3200 method: mercury species fractionation and quantification by microwave assisted extraction, selective solvent extraction and/or solid phase extraction. U.S. environmental protection agency http://www.epa.gov/osw/hazard/testmethods/pdfs/3200.pdf (accessed 01.05.2013)

[B20] Butcher DJ Review: recent advances in optical atomic spectrometry Appl Spectrosc Rev 2013 48 261 328 10.1080/05704928.2012.717570

[B21] Long Z Luo YM Zheng CB Deng PC Hou XD Recent advance of hydride generation-analytical atomic spectrometry: part I - technique development Appl Spectrosc Rev 2012 47 382 413 10.1080/05704928.2012.666775

[B22] Long Z Chen C Hou XD Zheng CB Recent advance of hydride generation-analytical atomic spectrometry: part II – analysis of real samples Appl Spectrosc Rev 2012 47 495 517 10.1080/05704928.2012.666776

[B23] Bings NH Bogaerts A Broekaert JAC Atomic spectroscopy Anal Chem 2013 85 670 704 10.1021/ac3031459 23134273

[B24] Cerveny V Horvath M Broekaert JAC Determination of mercury in water samples by electrochemical cold vapor generation coupled to microstrip microwave induced helium plasma optical emission spectrometry Microchem J 2013 107 10 16

[B25] Jamroz P Pohl P Zyrnicki W Spectroscopic evaluation of a low power atmospheric pressure mixed argon-helium microwave induced plasma combined with the chemical generation of volatile species for the optical emission spectrometric determination of arsenic, antimony and mercury J Anal At Spectrom 2012 27 1772 1779 10.1039/c2ja30063j

[B26] Frentiu T Mihaltan AI Darvasi E Ponta M Roman C Frentiu M A novel analytical system with a capacitively coupled plasma microtorch and a gold filament microcollector for the determination of total Hg in water by cold vapour atomic emission spectrometry J Anal At Spectrom 2012 27 1753 1760 10.1039/c2ja30156c

[B27] Frentiu T Mihaltan AI Ponta M Darvasi E Frentiu M Cordos E Mercury determination in non- and biodegradable materials by cold vapor capacitively coupled plasma microtorch atomic emission spectrometry J Hazard Mater 2011 193 65 69 21802847 10.1016/j.jhazmat.2011.07.031

[B28] Frentiu T Mihaltan AI Senila M Darvasi E Ponta M Frentiu M Pintican BP New method for mercury determination in microwave digested soil samples based on cold vapor capacitively coupled plasma microtorch optical emission spectrometry: comparison with atomic fluorescence spectrometry Microchem J 2013 110 545 552

[B29] Schollenberger CJ Determination of soil organic matter Soil Sci 1945 59 53 56 10.1097/00010694-194501000-00008

[B30] Levei E Frentiu T Ponta M Tanaselia C Borodi G Characterization and assessment of potential environmental risk of tailings stored in seven impoundments in the Aries river basin, Wester Romania Chem Cent J 2013 7 5 10.1186/1752-153X-7-5 23311708 PMC3558456

[B31] Abbollino O Malandrino M Giacomino A Mentasi E The role of chemometrics in single and sequential extraction assay: a review: part I. Extraction procedures, uni- and bivariate techniques and multivariate reduction techniques for pattern recognition Anal Chim Acta 2011 688 104 121 10.1016/j.aca.2010.12.020 21334476

[B32] Zhou F Guo H Liu Z Jiang Y Chemometrics data analysis of marine water quality and source identification in Southern Hong Kong Mar Pollut Bull 2007 54 745 756 10.1016/j.marpolbul.2007.01.006 17320914

[B33] Bland JM Altman DG Measuring agreement in method comparison studies Stat Methods Med Res 1999 8 135 160 10.1191/096228099673819272 10501650

[B34] Rytuba JJ Mercury from mineral deposits and potential environmental impact Environ Geol 2003 43 326 338

[B35] Kocman D Horvat M Kotnik J Mercury fractionation in contaminated soils from the Idrija mercury mine region J Environ Monitor 2004 6 696 703 10.1039/b403625e 15292953

[B36] Molina JA Oyarzun R Esbri JM Higueras P Mercury accumulation in soils and plants in the Almaden mining district, Spain; one of the most contaminated sites on Earth Environ Geochem Hlth 2006 28 487 498 10.1007/s10653-006-9058-9 17013679

[B37] Ministerial Order No. 756/1997 Approving the regulation concerning the assessment of environmental pollution Official Gazette 1997 Part I, no. 303bis/06.11.1997 [in Romanian]

[B38] Hakanson L Ecological risk index for aquatic pollution control. A sedimentological approach Water Res 1995 14 975 1001

[B39] Ministerial Order No. 95/2005 for the approval of waste acceptance criteria and national list of waste accepted in each landfill class Official Gazette 2005 Part I, no. 194/08.03.2005 [in Romanian]

[B40] Shao D Kang Y Wu S Wong MH Effects of sulfate reducing bacteria and sulfate concentrations on mercury methylation in freshwater sediments Sci Total Environ 2012 424 331 336 22444059 10.1016/j.scitotenv.2011.09.042

[B41] Kinniburg GG Jackson ML Absorption of mercury (II) by iron hydrous oxide gel Soil Sci Soc Am J 1978 42 45 47 10.2136/sssaj1978.03615995004200010010x

[B42] Geffery PG Chemical methods of rock analysis 1975 2 Oxford: Pergamon

[B43] Renneberg AJ Dudas MJ Calcium hypochlorite removal of mercury and petroleum hydrocarbons from co-contaminated soils Waste Manage Res 2002 20 468 475 10.1177/0734242X0202000510 12498483

