# Supplementary material for: Determination, speciation and distribution of mercury in soil in the surroundings of a former chlor-alkali plant: assessment of sequential extraction procedure and analytical technique
Source: Chem Cent J. 2013 Nov 19;7:178. doi: 10.1186/1752-153X-7-178 (PMC4176730; doi:10.1186/1752-153X-7-178)
Supplement: Additional file 1 — Characteristics and working conditions of the CV-μCCP-OES analytical system. [file 1752-153X-7-178-S1.doc]

**Additional file 1** -Characteristics and working conditions of the CV-μCCP-OES analytical system

| **Component** | **Characteristic/operating conditions** |
| --- | --- |
| Plasma microtorch (home-made,  INCDO-INOE 2000 Bucharest, Research Institute for Analytical Instrumentation, Cluj-Napoca, Romania) | Capacitively coupled with 1.25 mm microelectrode of Mo (Goodfellow, Cambridge, UK) mounted in a Mo support inside a quartz tube (25 mm length, 5 mm i.d, 160 nm cut-off; H. Baumbach & Co, Ltd., Ipswich Suffolk, UK).  Ar 5.0 quality to sustain plasma, 150 ml/ min flow rate  Plasma power: 10 W. |
| Plasma power supply (Technical University, Cluj-Napoca, Romania) | Free-running generator (13.56 MHz, 10 – 40 W). Size LxWxH: 15x17x24 cm^3^ |
| Ocean Optics Microspectrometer (Dunedin, Florida, USA) | QE65 Pro (190 – 350 nm spectral range, 0.4 nm FWHM, back illuminated Hamamatsu S7031-1006S detector, Peltier cooled detector (–20 ºC). |
| CETAC cold vapor generator (Omaha, Nebraska, USA) | HGX-200 model equipped with a 4-channel peristaltic pump. Sample flow rate: 3.5 ml/min, SnCl_2_ solution flow rate: 1 ml/min. |
| Data acquisition and signal processing | Spectrasuite software, background correction, 10 s integration time. |

.
