# Supplementary material for: Determination, speciation and distribution of mercury in soil in the surroundings of a former chlor-alkali plant: assessment of sequential extraction procedure and analytical technique
Source: Chem Cent J. 2013 Nov 19;7:178. doi: 10.1186/1752-153X-7-178 (PMC4176730; doi:10.1186/1752-153X-7-178)
Supplement: Additional file 2 — Chemical characterization of soil. [file 1752-153X-7-178-S2.doc]

**Additional file 2 – Chemical characterization of soil**

| **Sample** | **pH** | **TOC^a^**  **(%)** | **Total metal content** | | | | | | | | | | | | | | | **Water leachable content (mg/kg)^b^** | | |
| --- | --- | --- | --- | --- | --- | --- | --- | --- | --- | --- | --- | --- | --- | --- | --- | --- | --- | --- | --- | --- |
|  |  |  | **(%)** | | | | | | |  | | **(mg/kg)^b^** | | | | | |  |  |  |
|  |  |  | **Al** | **Ca** | **Fe** | **K** | **Mg** | **Mn** | **Na** |  | | **Ba** | **Cr** | **Cu** | **Li** | **Sr** | **Zn** | **Cl^-^** | **NO_3_^-^** | **SO_4_^2-^** |
| 1 | 8.0 | 2.78 | 2.72 | 2.70 | 2.75 | 1.51 | 0.69 | 0.073 | 0.078 | | 197 | | 28.4 | 51.7 | 55.3 | 189 | 156 | 21.5 | 43.5 | 19.2 |
| 2 | 9.1 | 1.29 | 1.48 | 1.82 | 2.49 | 0.63 | 0.54 | 0.071 | 0.044 | | 116 | | 45.4 | 48.5 | 21.7 | 63 | 146 | 3.02 | 3.34 | 23.0 |
| 3 | 8.1 | 0.24 | 1.23 | 4.56 | 1.95 | 0.48 | 0.51 | 0.069 | 0.060 | | 160 | | 2.0^c^ | 165 | 28.3 | 168 | 590 | 14.0 | 150 | 23.4 |
| 4 | 9.3 | 0.55 | 1.14 | 2.76 | 3.15 | 0.55 | 0.61 | 0.084 | 0.033 | | 131 | | 32.5 | 54.1 | 22.9 | 114 | 208 | 3.33 | 6.69 | 24.9 |
| 5 | 8.9 | 1.52 | 1.93 | 4.58 | 2.86 | 1.14 | 0.97 | 0.086 | 0.065 | | 86.1 | | 48.2 | 45.9 | 53.2 | 199 | 139 | 3.41 | 149 | 13.5 |
| 6 | 9.2 | 1.35 | 1.21 | 6.26 | 1.98 | 0.77 | 0.48 | 0.068 | 0.044 | | 174 | | 39.2 | 67.7 | 15.3 | 137 | 303 | 6.58 | 36.3 | 30.1 |
| 7 | 8.3 | 1.31 | 1.52 | 4.15 | 2.15 | 1.03 | 0.54 | 0.086 | 0.056 | | 178 | | 51.1 | 58.0 | 23.6 | 143 | 180 | 7.41 | 2.44 | 38.6 |
| 8 | 8.3 | 1.46 | 1.52 | 3.34 | 2.07 | 0.79 | 0.50 | 0.072 | 0.066 | | 152 | | 55.9 | 85.9 | 16.1 | 137 | 200 | 6.89 | 24.9 | 31.3 |
| 9 | 8.8 | 1.68 | 1.29 | 6.01 | 2.07 | 0.69 | 0.48 | 0.065 | 0.087 | | 160 | | 36.7 | 76.4 | 91.2 | 108 | 366 | 2.71 | 0.19 | 19.1 |
| 10 | 8.6 | 1.32 | 1.60 | 3.95 | 2.58 | 0.93 | 0.66 | 0.075 | 0.048 | | 169 | | 48.1 | 66.0 | 30.2 | 141 | 253 | 3.53 | 0.20 | 25.1 |
| 11 | 8.5 | 0.85 | 1.19 | 2.39 | 2.35 | 0.48 | 0.60 | 0.075 | 0.026 | | 112 | | 33.6 | 47.3 | 23.7 | 58 | 162 | 4.84 | 3.29 | 24.2 |
| 12 | 8.9 | 0.29 | 0.81 | 5.38 | 1.69 | 0.56 | 0.48 | 0.069 | 0.041 | | 127 | | 32.6 | 57.5 | 11.0 | 137 | 166 | 9.03 | 0.19 | 14.2 |
| 13 | 8.8 | 1.64 | 1.06 | 5.59 | 1.98 | 0.48 | 0.47 | 0.053 | 0.058 | | 84.7 | | 42.6 | 90.3 | 9.56 | 97 | 236 | 7.75 | 2.70 | 48.7 |
| 14 | 8.0 | 1.26 | 1.72 | 4.05 | 2.84 | 0.93 | 0.97 | 0.079 | 0.050 | | 82.7 | | 33.3 | 46.0 | 50.9 | 176 | 117 | 4.59 | 2.64 | 14.9 |
| 15 | 8.1 | 0.33 | 2.43 | 3.99 | 2.57 | 0.56 | 0.62 | 0.082 | 0.060 | | 225 | | 2.0^c^ | 158 | 28.4 | 128 | 798 | 79.6 | 93.5 | 338 |
| 16 | 8.0 | 1.07 | 1.31 | 3.01 | 4.58 | 0.56 | 0.51 | 0.074 | 0.082 | | 240 | | 45.8 | 478 | 21.7 | 103 | 418 | 45.7 | 37.7 | 1339 |
| 17 | 8.3 | 1.20 | 1.21 | 2.91 | 2.99 | 0.41 | 0.48 | 0.067 | 0.090 | | 127 | | 11.3 | 127 | 27.4 | 110 | 223 | 7.19 | 89.9 | 370 |
| 18 | 8.3 | 0.33 | 0.83 | 6.58 | 1.52 | 0.36 | 0.45 | 0.045 | 1.24 | | 78.3 | | 5.25 | 54.7 | 16.6 | 140 | 77 | 12388 | 94.7 | 1405 |
| 19 | 8.2 | 0.47 | 1.20 | 7.25 | 2.32 | 0.52 | 0.49 | 0.058 | 0.10 | | 168 | | 17.9 | 294 | 21.3 | 114 | 234 | 4.04 | 46.4 | 151 |
| 20 | 8.5 | 0.13 | 1.00 | 5.27 | 2.35 | 0.37 | 0.52 | 0.073 | 0.068 | | 122 | | 11.7 | 120 | 27.1 | 127 | 180 | 217 | 71.0 | 187 |
| 21 | 8.4 | 1.19 | 1.39 | 9.24 | 2.36 | 0.66 | 0.65 | 0.075 | 0.12 | | 151 | | 24.5 | 209 | 33.5 | 174 | 598 | 49.1 | 21.2 | 339 |
| 22 | 8.2 | 1.77 | 0.86 | 13.4 | 1.67 | 0.59 | 0.39 | 0.045 | 0.19 | | 92.0 | | 17.3 | 111 | 34.0 | 163 | 76 | 363 | 40.8 | 336 |
| 23 | 8.9 | 1.28 | 1.06 | 3.15 | 2.01 | 0.58 | 0.44 | 0.069 | 0.029 | | 139 | | 42.3 | 75.4 | 10.7 | 90 | 171 | 152 | 61.8 | 203 |
| 24 | 8.7 | 1.49 | 0.55 | 3.81 | 1.95 | 0.19 | 0.30 | 0.057 | 0.051 | | 92.7 | | 58.3 | 80.7 | 10.5 | 71 | 211 | 5.69 | 6.86 | 43.3 |
| 25 | 8.3 | 1.25 | 1.06 | 2.27 | 2.27 | 0.56 | 0.53 | 0.063 | 0.032 | | 101 | | 31.5 | 80.0 | 18.8 | 76 | 188 | 2.52 | 0.14 | 21.3 |
| 26 | 8.8 | 1.65 | 1.28 | 3.25 | 2.53 | 0.60 | 0.49 | 0.063 | 0.056 | | 138 | | 53.3 | 70.1 | 13.4 | 81 | 241 | 9.47 | 0.19 | 58.7 |
| 27 | 7.7 | 0.68 | 1.58 | 1.15 | 2.79 | 0.61 | 0.59 | 0.067 | 0.033 | | 127 | | 55.3 | 48.8 | 24.6 | 51 | 145 | 3.92 | 1.96 | 14.7 |
| 28 | 8.4 | 0.81 | 1.29 | 0.97 | 3.10 | 0.44 | 0.44 | 0.12 | 0.023 | | 139 | | 40.3 | 40.7 | 8.03 | 45 | 111 | 6.42 | 0.24 | 88.3 |
| 29 | 8.1 | 1.64 | 1.16 | 2.84 | 2.16 | 0.50 | 0.54 | 0.060 | 0.038 | | 95.2 | | 35.6 | 67.1 | 13.4 | 61 | 182 | 2.94 | 13.7 | 24.3 |
| 30 | 8.4 | 1.40 | 1.97 | 1.28 | 2.54 | 0.54 | 0.59 | 0.036 | 0.043 | | 156 | | 51.9 | 45.8 | 27.4 | 49 | 139 | 3.49 | 0.19 | 22.9 |
| 31 | 7.8 | 1.01 | 1.26 | 0.94 | 2.79 | 0.49 | 0.34 | 0.14 | 0.021 | | 133 | | 44.4 | 31.3 | 6.31 | 43 | 108 | 2.79 | 0.20 | 13.3 |
| 32 | 8.2 | 1.55 | 1.17 | 2.00 | 2.50 | 0.45 | 0.58 | 0.10 | 0.024 | | 106 | | 24.1 | 63.9 | 14.4 | 50 | 212 | 3.33 | 0.20 | 15.1 |
| 33 | 8.2 | 1.44 | 1.51 | 1.04 | 3.15 | 0.49 | 0.41 | 0.096 | 0.026 | | 145 | | 47.2 | 31.8 | 10.0 | 43 | 93 | 3.79 | 0.60 | 13.2 |
| 34 | 8.5 | 0.15 | 2.10 | 0.95 | 2.64 | 0.50 | 0.41 | 0.065 | 0.055 | | 150 | | 2.0^c^ | 203 | 28.1 | 138 | 565 | 3.43 | 273 | 35.8 |
| 35 | 8.5 | 0.29 | 1.02 | 1.95 | 2.29 | 0.38 | 0.57 | 0.076 | 0.055 | | 107 | | 3.33 | 87.2 | 14.7 | 71 | 203 | 8.49 | 128 | 41.6 |
| 36 | 8.3 | 1.02 | 1.41 | 6.70 | 1.30 | 1.37 | 0.47 | 0.027 | 0.080 | | 122 | | 18.2 | 114 | 33.7 | 208 | 500 | 111 | 129 | 402 |
| 37 | 8.4 | 2.41 | 1.31 | 1.48 | 2.45 | 0.41 | 0.61 | 0.12 | 0.040 | | 89.1 | | 13.9 | 288 | 25.9 | 87 | 274 | 18.5 | 12.0 | 843 |
| 38 | 8.4 | 2.20 | 0.93 | 2.30 | 2.21 | 0.14 | 0.49 | 0.059 | 0.059 | | 96.9 | | 2.0^c^ | 197 | 28.1 | 138 | 505 | 14.4 | 260 | 78.1 |
| **Mean** | **8.4** | **1.17** | **1.35** | **3.82** | **2.42** | **0.61** | **0.54** | **0.073** | **0.089** | | **133** | | **34.7** | **106** | **25.0** | **111** | **255** | **358** | **47.6** | **177** |
| **Variance** | **0.4** | **0.63** | **0.44** | **2.56** | **0.57** | **0.28** | **0.13** | **0.022** | **0.19** | | **38.5** | | **15.4** | **90.5** | **16.1** | **47.4** | **169** | **2006** | **69.4** | **331** |
| **Median** | **8.4** | **1.26** | **1.26** | **3.20** | **2.362** | **0.55** | **0.51** | **0.070** | **0.055** | | **129** | | **35.2** | **75.9** | **23.2** | **111** | **202** | **7.04** | **17.5** | **37.2** |
| **Min** | **7.7** | **0.13** | **0.13** | **0.94** | **1.30** | **0.14** | **0.30** | **0.027** | **0.021** | | **78.3** | | **3.33^c^** | **31.3** | **6.31** | **43.0** | **76.0** | **2.52** | **0.14** | **13.2** |
| **Max** | **9.3** | **2.78** | **2.78** | **13.4** | **4.58** | **1.51** | **0.97** | **0.14** | **1.24** | | **240** | | **58.3** | **478** | **91.2** | **208** | **798** | **12388** | **273** | **1405** |

^a^ – total organic carbon

^b^ - n = 3 complete dissolution/analysis sequences for each sample

^c^ – limit of detection
