# Supplementary figures and images for: Determination, speciation and distribution of mercury in soil in the surroundings of a former chlor-alkali plant: assessment of sequential extraction procedure and analytical technique
Source: Chem Cent J. 2013 Nov 19;7:178. doi: 10.1186/1752-153X-7-178 (PMC4176730; doi:10.1186/1752-153X-7-178)

**Additional file 3 – Two dimensional plot of PCs considering all investigated parameters of soil**


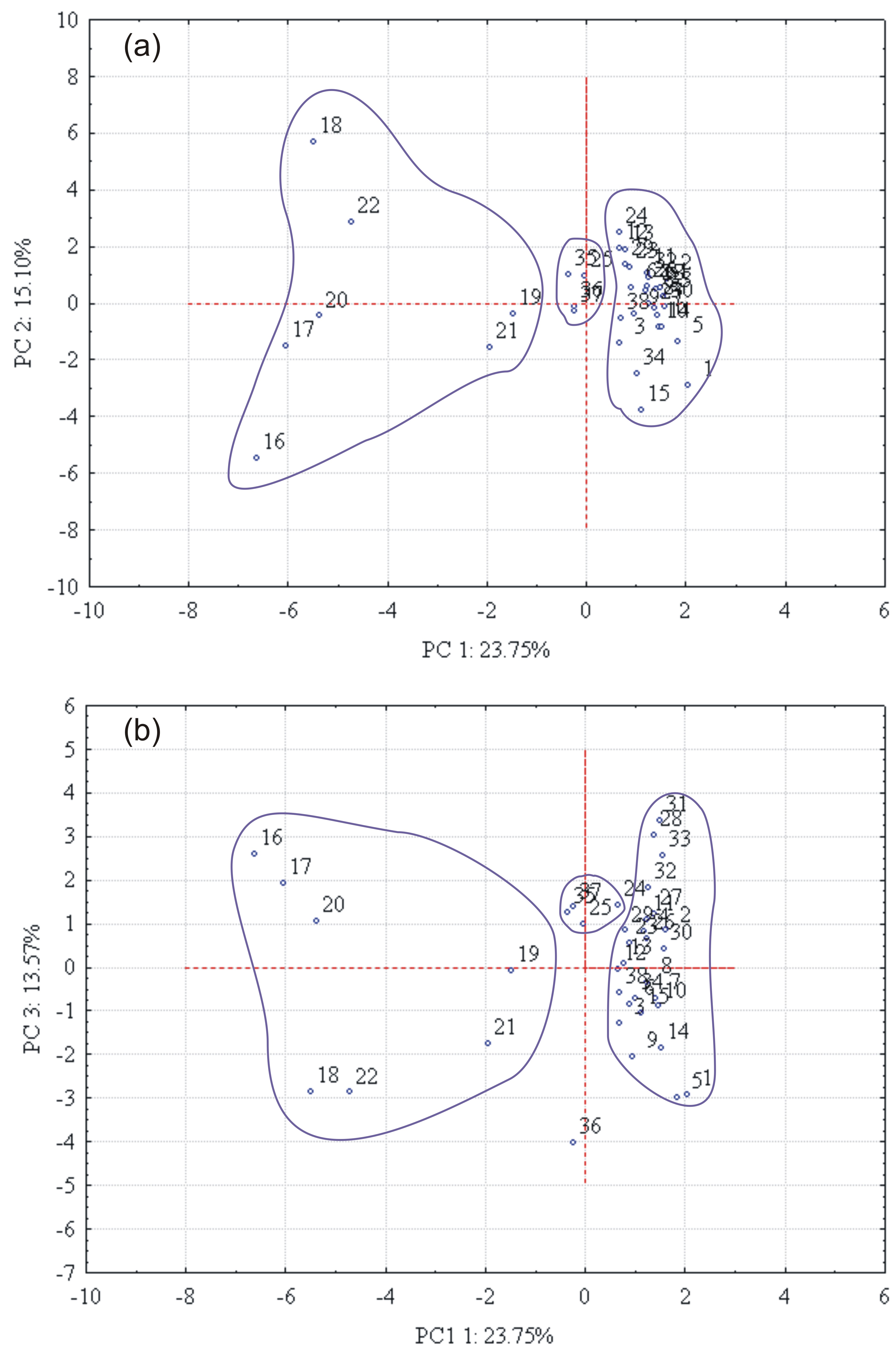

Supplement: Additional file 3 — Two dimensional plot of PCs considering all investigated parameters of soil. [file 1752-153X-7-178-S3.doc]

**Additional file 4 – Two dimensional plot of PCs considering Hg parameters of soil**


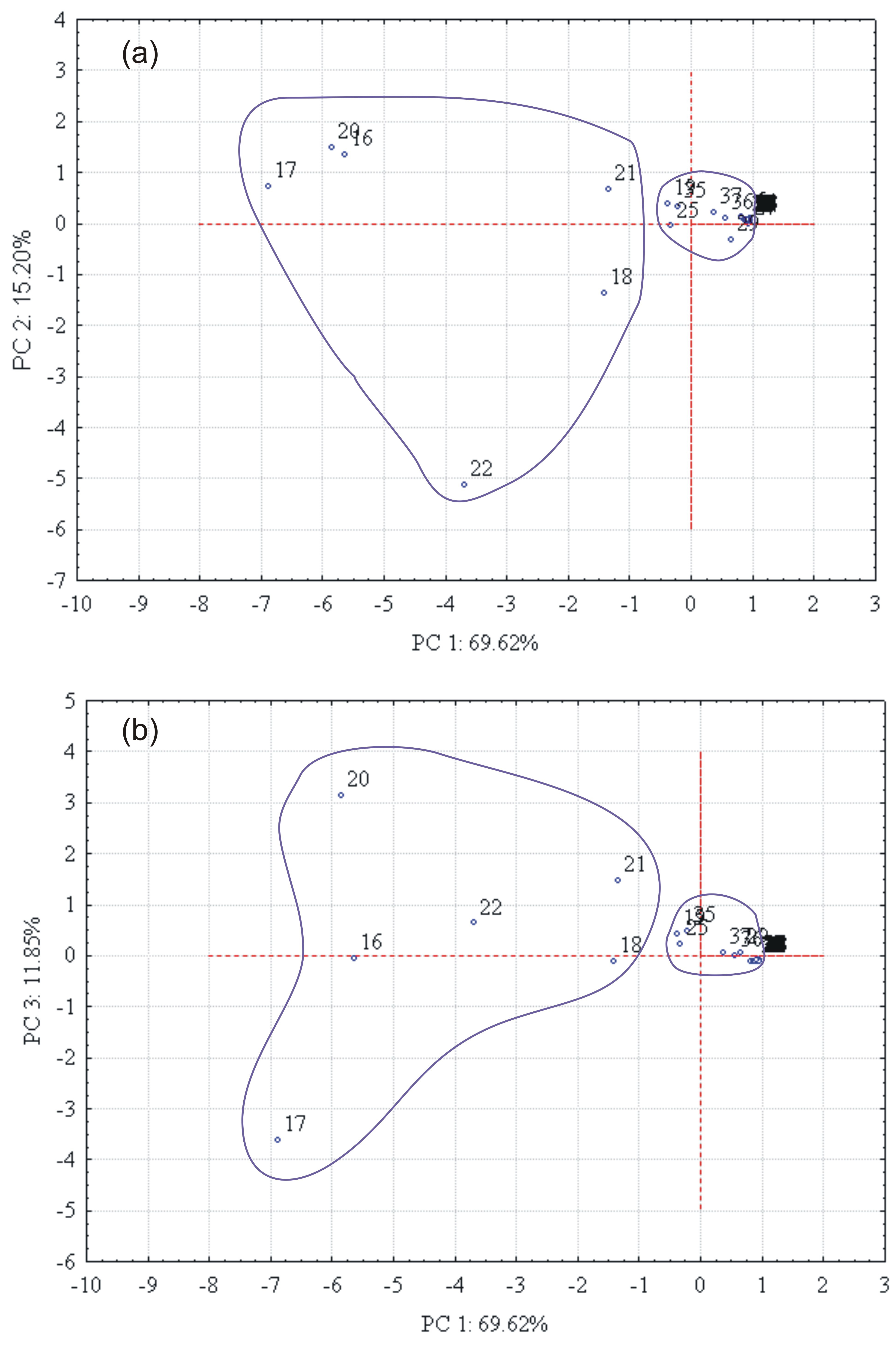

Supplement: Additional file 4 — Two dimensional plot of PCs considering Hg parameters of soil. [file 1752-153X-7-178-S4.doc]
